# Inverse Design and Numerical Investigations of an Ultra-Compact Integrated Optical Switch Based on Phase Change Material

**DOI:** 10.3390/nano13101643

**Published:** 2023-05-15

**Authors:** Kun Yin, Yang Gao, Hao Shi, Shiqiang Zhu

**Affiliations:** 1 School of Mechanical Engineering, Zhejiang University, Hangzhou 310007, China; 2 Zhejiang Lab, Hangzhou 311112, China

**Keywords:** integrated optics, optical switch, phase change material, silicon photonics

## Abstract

The miniaturization of optical switches is a promising prospect with the use of phase-change materials (PCMs), and exploring various strategies to effectively integrate PCMs with integrated optical waveguides represents an intriguing research question. In this study, an ultra-compact integrated optical switch based on PCM is proposed. This device consists of a Ge${}_{2}$Sb${}_{2}$Te${}_{5}$ nano-disk and an inverse-designed pixelated sub-wavelength structure. The pixelated sub-wavelength structure offers customized refractive indices that conventional materials or structures cannot achieve, leading to an improved insertion loss (IL) and extinction ratio (ER) performance of the device. Furthermore, this structure enhances the interaction between the optical field and GST, resulting in a reduction of the device size and the inserted GST footprint. With an ultra-compact device footprint of 0.9 µm × 1.5 µm, the simulation results exhibit a low IL of 0.45 dB, and a high ER of 18.0 dB at 1550 nm. Additionally, relevant studies show that this device is able to perform reliably despite minor variations in the manufacturing process.

## 1. Introduction

Recent research has demonstrated that large-scale photonic integrated circuits (PICs) based on silicon exhibit exceptional properties such as high bandwidth, ultra-fast transmission rates, and superior anti-interference capabilities, making them highly attractive for meeting the growing demands of high-speed optical interconnection in datacenter networks and optical deep learning [1,2,3,4,5]. Despite significant research efforts and rapid development in recent years, PICs still significantly lag behind electrical integrated circuits (EICs) in terms of integration level, which has resulted in a growing demand for the miniaturization of photonic devices. An essential element of PICs is an optical switch with compact footprint, high performance and good machining tolerances—a challenging proposition for traditional thermo-optic or free carrier switches.

Recently, phase change materials (PCMs) have been extensively investigated to tackle the challenge of miniaturizing active devices in PICs due to their unique ability to undergo reversible conversion between states and exhibit significant refractive index contrast [6,7,8,9]. The phase transition in PCMs can be triggered by various stimuli, including heat, electricity or optics, leading to apparent differences in their electrical and optical properties during and after the phase transition [10]. Transition metal oxides or semiconductor oxides, such as VO${}_{2}$, and chalcogenide compounds, such as Ge${}_{2}$Sb${}_{2}$Te${}_{5}$ (GST), are two types of PCMs that have been extensively investigated. VO${}_{2}$ undergoes a structural shift from a monoclinic insulating phase to a tetragonal metal phase at temperatures above 68 ${}^{\circ }$C, resulting in a strong transition from low to high absorption in the mid-infrared band [11,12,13]. Ge${}_{2}$Sb${}_{2}$Te${}_{5}$ (GST), on the other hand, exhibits significant changes in refractive and absorption indices during its transition from the crystalline to amorphous state. Moreover, GST can maintain a specific state without energy supply, making it highly favourable for non-volatile memory applications [14,15,16,17,18].

Despite the unique ability of PCMs to exhibit significant refractive index contrast between states, the real and imaginary parts of the refractive index of PCMs undergo changes simultaneously in their different states. As a result, the wavefront and propagation losses of light in PCMs also concomitantly vary with the state transition, which can lead to a deterioration of device performance. For example, one possible integration of PCMs with integrated optical switches is using PCM thin films to block light propagation directly based on their loss absorption properties, taking advantage of the significant refractive index contrast exhibited by PCMs. However, this configuration poses certain limitations, such as a limited extinction ratio (ER) below 10 dB and a relatively large size [19]. Another strategy is to increase the volume of the PCM cell deposited upon waveguides; however, it degrades the operation speed and the repeatability of the phase change process [20]. Thus, the challenge remains in exploring various strategies to effectively integrate PCMs with integrated optical waveguides to improve device performance.

Recently, researchers have demonstrated that hyperbolic metamaterials consisting of alternating VO${}_{2}$/Si thin layers could significantly reduce effective optical absorptivity when VO${}_{2}$ is in the tetragonal metallic phase [21]. Consisting of only 20-pair hyperbolic metamaterials, this device ensures a good ER, a compact footprint of 400 nm × 220 nm × 200 nm (width × height × length), the modulation depth of 5.6 dB and low insertion loss(IL) of 1.25 dB. In 2022, Wu proposed a new approach to ultra-compact on-chip switches based on structured PCMs [19]. Three GST nano-disks with a total footprint of 0.229 µm${}^{2}$× 35 nm on top of a single mode strip silicon waveguide, which were specifically distributed by intelligent algorithm, were used to realize ultra-compacted optical switches with a high ER up to 27 dB.

In this paper, to effectively integrate PCMs with integrated optical switch waveguides and improve device performance, a novel ultra-compact integrated optical switch based on PCM is proposed, which is designed with one GST nano-disk and a inverse-designed pixilated sub-wavelength structure. On the one hand, the pixelated sub-wavelength structure is an engineered artificial structure with controllable electromagnetic properties that are unattainable in conventional materials or structures [22,23,24]. This structure scatters and absorbs incident light with a customizable refractive index, leading to a significant decrease in the overall IL and an improvement in the ER of the device. On the other hand, the pixelated sub-wavelength structure enhances the interaction between the optical field and GST. As a result, both the device size and the inserted GST footprint decrease, leading to improvements in the operation speed and repeatability of the phase-change process.

## 2. Materials and Methods

### 2.1. Device Structure and Working Principle

**Figure 1:**
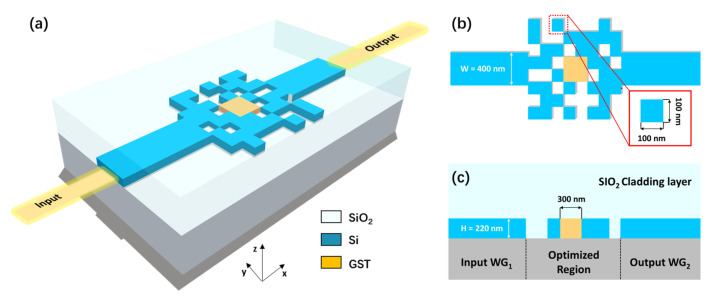
Schematic diagram of the proposed ultra-compact optical switch. (**a**) Three-dimensional perspective view of the optical switch. (**b**) Top view of the device, the inset shows the zoom-in view of the pixel. (**c**) Cross-sectional view of the device in xy-plane. Note that the schematic view indicates a general layout and is not to scale.

The schematic of the proposed on-chip switch is shown in Figure 1. The device is designed on the most common silicon-on-insulator (SOI) substrate with 220-nm-thick top silicon and 2-µm-thick buried oxide. A 2-µm-thick SiO${}_{2}$ is deposited on the silicon layer as the upper cladding layer. The widths of the input waveguide and output waveguide are both 400 nm to support just the fundamental TE mode. The rectangular optimized region is discretized into M × N square units, called “pixels”, with each pixel having a side width of 100 nm and a depth of 220 nm. A square area at the center of the optimized region, consisting of 3 × 3 pixels, is fully filled by GST. At 1550 nm, when GST is in the amorphous state (a-GST), the corresponding refractive index is 3.98 + 0.0244i [20]. The real part of the refractive index is close to that of the silicon and the imaginary part is low, indicating low absorption. At this time, the IL of the device is low and the switch state is ON. When GST changes to the crystalline state (c-GST) with its refractive index increased to 6.49 + 1.054i [20], contrarily, the switch state turns OFF. Unless specified otherwise, the “ypixel” or “pixels” mentioned below refers to the pixels not filled with GST material. Each of the remaining pixels is occupied with silicon or silica. The arrangement of these pixels will affect the distribution of effective refractive indices and therefore manipulates the evolution of light field. Our goal is to reasonably arrange the material distribution of the optimized region to maximize the transmittance of input TE${}_{0}$ mode when GST is in the amorphous state, while simultaneously minimizing the transmittance when GST is in the crystalline state, that is to say, minimizing IL and maximizing the ER of the on-chip switch.

### 2.2. Inverse Design Method

In recent years, the inverse design method and direct binary search (DBS) algorithm have emerged as powerful tools to create free-form metamaterials that enable the design of ultra-compact and highly functional devices [25,26]. Unlike traditional device design methods, the inverse design approach allows for the flexible engineering of refractive index distribution which can be leveraged to realize a wide range of integrated devices with ultra-compact form factors. Building upon this advancement, we utilized the DBS algorithm to determine the material properties of each pixel in our proposed design. Although the DBS optimization algorithm can only find the local optimal solution, it has the advantage that it is guaranteed to converge to a solution in any case. Notably, the optimization result is usually sensitive to the initial patterns. In this paper, we manually set the inverse design region as the all-silicon initial structure at the beginning. To evaluate the device performance during the optimization process, the figure-of-merit (FOM) is defined as:

(1) $$FOM=\frac{1}{M}\sum \left(t_{ai}-t_{ci}\right)$$

where $t_{ai}$ and $t_{ci}$ are the transmittances of input TE${}_{0}$ mode when GST is in the amorphous state or in the crystalline state at the i-th wavelength channel within the wavelength range from 1535 nm to 1565 nm. *M* denotes the number of wavelength steps and *M* is chosen to be 30 in this design. For an ideal switch device, $t_{a}$−$t_{c}$ should be 1, corresponding to FOM = 1. A 3D finite-difference time-domain (FDTD) method is utilized to calculate the FOM via commercial software (Lumerical FDTD Solutions). In each iteration, we switch the material property in each pixel in turn (Si to SiO${}_{2}$ or SiO${}_{2}$ to Si), and then calculate the FOM. If the FOM is improved, the new material state of the pixel will be maintained. If not, the pixel returns to its original state, and the algorithm proceeds to the next pixel. One iteration ends after all the pixel states are inspected. Then the iterations continue until the FOM exhibits no great improvement (<1% for our case).

## 3. Results

### 3.1. Numerical Evaluation

**Figure 2:**
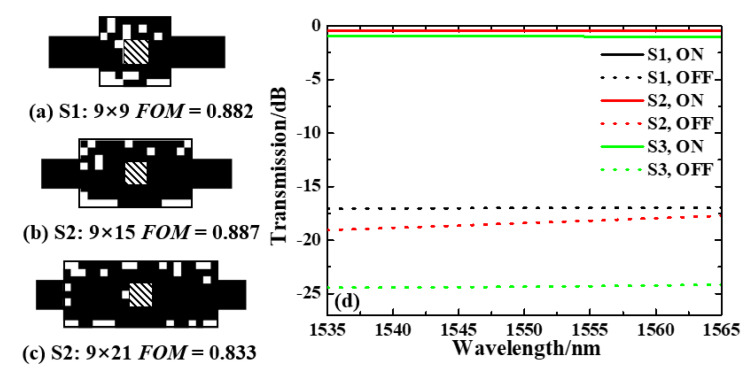
Optimized results for the different footprints of the optimized regions. The final optimized pattern of the optical switch and corresponding FOM of (**a**) S1, (**b**) S2, (**c**) S3. (**d**) Normalized calculated transmission spectra for S1, S2 and S3 within the wavelength range from 1535 nm to 1565 nm.

The selection of the optimization area footprint is subjective in the DBS algorithm. As a comparison, we optimized the device with different footprints of the optimized regions of 0.9 × 0.9 µm${}^{2}$ (S1), 0.9 × 1.5 µm${}^{2}$ (S2), and 0.9 × 2.1 µm${}^{2}$ (S3), respectively. The final optimized patterns of S1, S2 and S3 and corresponding FOMs are shown in Figure 2a–c. The normalized calculated transmission spectra for S1, S2 and S3 as a function of wavelength are shown in Figure 2d. Table 1 provides a detailed summary of the IL and ER performance of the proposed switches at 1550 nm, as well as their ranges over the C band. As illustrated in Figure 2d, the transmission spectra of all three switches are wavelength-insensitive. Among them, switch S1 exhibits a maximum IL and ER of 0.48 dB and 16.5 dB, respectively, at 1565 nm. On the other hand, switch S2 demonstrates an IL of less than 0.47 dB and an ER greater than 17.3 dB across the C band, with a low IL of 0.45 dB and an ER of approximately 18.0 dB at 1550 nm. While switch S3 outperforms both switches S1 and S2 in terms of ER, its IL is greater than 0.95 dB from 1535 nm to 1565 nm. Considering the device footprint, IL and ER characteristics of the three switches, it is apparent that S2 is the best performer. Subsequently, we will focus our discussion on switch S2.

**Figure 3:**
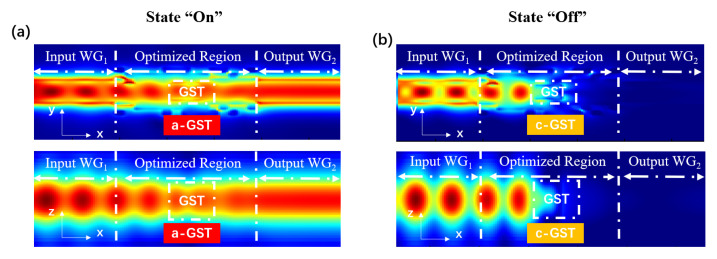
Simulated electric-field intensity distributions of S2 in the *x*-*y* and the *x*-*z* axis for the (**a**) ON state (a-GST) and the (**b**) OFF state (c-GST).

Figure 3 presents simulated electric-field intensity distributions in S2 at 1550 nm. As shown in Figure 3a, the input light continues to propagate along the waveguide with an acceptable attenuation (≈0.45 dB) after passing through the region of the a-GST. On the other hand, as shown in Figure 3b, the input light is quickly extinct by the c-GST region and drops to nearly zero. Within the wavelength range 1530–1565 nm, ILs of the device vary from 1.1 to 1.2 dB and ERs vary from 14.2 to 17.6 dB. The application of the inverse design algorithm changes the effective refractive index distribution of the optimized area, which significantly improved the performance of the switch.

### 3.2. Experimental Feasibility

**Figure 4:**
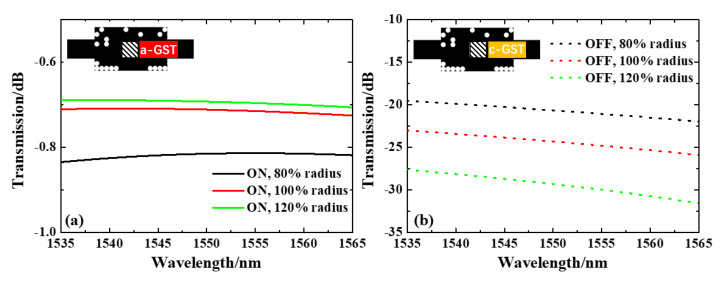
Normalized transmission spectra under uniform radius variation of (**a**) a-GST and (**b**) b-GST.

Performances of sub-wavelength structure devices can be greatly affected by process errors. To verify the practical application of the integrated optical switch we designed, we evaluated its process tolerance. The minimum line width of the micro-structure pixel is 100 nm, and a roundness effect may occur in the actual processing. Thus, we set the structure of the pixel as a circle to simulate this effect, while changing the radius of the circular hole to simulate line width variation. As shown in Figure 4, with uniform roundness effect, as the SiO${}_{2}$ pixels radius shift −20% to +20% from the original value (50 µm), the overall IL at 1535 nm–1565 nm is below 0.9 dB in the ON state, and the overall transmission drops to >18 dB in the OFF state. At 1550 nm, the ILs are 0.81, 0.71 and 0.69 dB, and the ERs are 19.8 dB, 23.6 dB and 28.6 dB for −20%, 0, and +20% variation, respectively, from the original radius 50 µm. This simulation result validates that this design is tolerant to uniform roundness effect.

**Figure 5:**
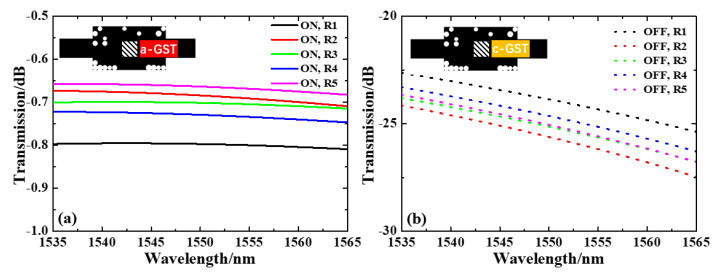
Normalized transmission spectra under random radius variation of (**a**) a-GST and (**b**) b-GST.

In a real-world process, the roundness effects are subject to random changes. Thus, a statistical evaluation of random radius configuration is simulated; the results are shown in Figure 5. The radius of SiO${}_{2}$ pixels randomly varies within the range of −20% to +20% from the original value (50 µm) in configurations R1–R5. The overall IL at 1535–1565 nm is below 0.85 dB in the ON state, and the overall transmission drops to >22.5 dB in the OFF state. At 1550 nm, the ILs are 0.80, 0.68, 0.70, 0.73 and 0.66 dB, and the ERs are 23.1, 24.9, 24.4, 23.9 and 24.4 dB for −20%, 0, and +20% variation from configuration R1–R5, respectively. These IL and ER simulation results of random roundness error fall between the maximum and minimum value of the results of uniform roundness error, validating that this design is also tolerant to the random roundness effect.

**Figure 6:**
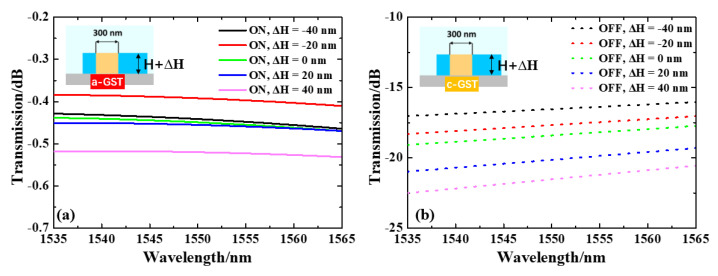
Normalized transmission spectra under under-fill and over-fill conditions of (**a**) a-GST and (**b**) b-GST.

In addition, under-fill or over-fill may occur when the hole is filled with evaporated GST, and we simulated the effect of this error by changing the height of the GST, as shown in Figure 6. The overall IL at 1535–1565 nm is below 0.53 dB in the ON state, and the overall transmission drops to >15 dB in the OFF state. For GST thickness varying −40 nm, −20 nm, 0, +20 nm and +40 nm from the original value, the ILs are 0.44, 0.39, 0.45, 0.46 and 0.52 dB, and the ERs are 16.1, 17.3, 18.0, 19.7 and 21.0 dB, respectively. These results validate that our design is robust to under-fill or over-fill error.

**Figure 7:**
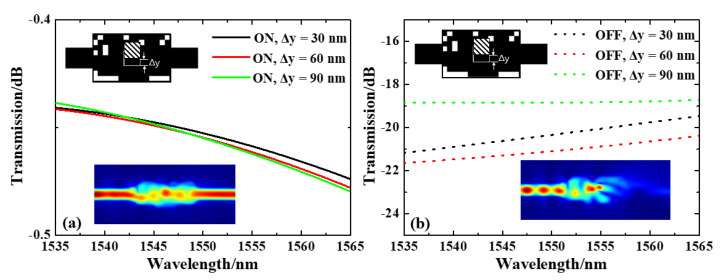
(**a**) Simulated electric-field intensity distributions and (**b**) Normalized calculated transmission spectra under deviation in different degrees.

To further evaluate the impact of offset error on device performance, we shifted the position of GST in the silicon sub-wavelength structure along the y direction by 30 nm, 60 nm, and 90 nm in the simulation. The results are shown in Figure 7. The overall IL of the device design remained largely unaffected by different offset errors, as confirmed by our investigation. Specifically, for offsets of 30 nm, 60 nm and 90 nm, the IL at 1550 nm remained consistent with a value of approximately 0.45 dB. The ER of 1550 nm for offset 30 nm, 60 nm and 90 nm are 19.9, 20.6 and 18.4 dB, respectively. When the offset error was less than 90 nm, the ER could still be guaranteed to be greater than 18.0 dB (ER of 0 y offset), which is considered within the usable range. Thus, we conclude that our design has a tolerance of approximately 90 nm for the lithography process. These findings provide valuable insights into optimizing fabrication processes for our proposed integrated optical switch, particularly in terms of lithography precision.

**Figure 8:**
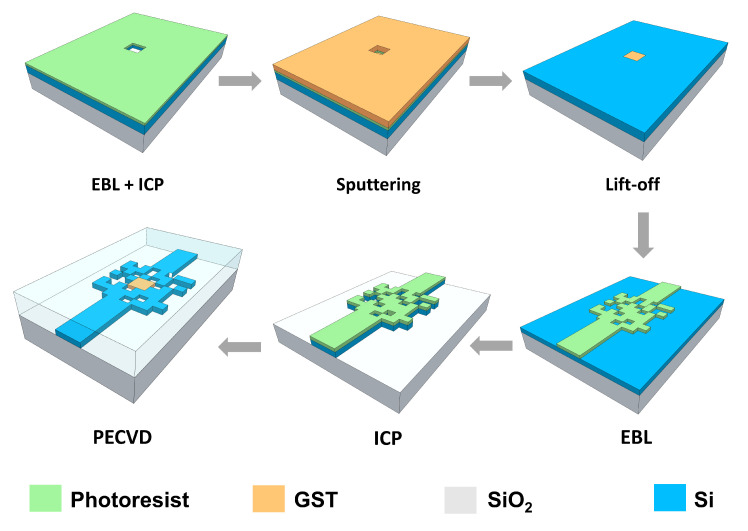
Fabrication flow schematics of the proposed ultra-compact optical switch based on GST material on SOI.

Presenting the fabrication flow schematics as shown in Figure 8 is aimed at providing a processing plan for replicating our proposed device structure, despite the fact that our design is based on numerical simulations. The device is fabricated on an SOI wafer with a 220-nm-thick silicon layer on top of a 2-µm-thick buried oxide layer. At first, the GST window is patterned by electron-beam lithography (EBL) and inductively coupled plasma (ICP). A 220 nm thick GST layer was deposited using a magnetron sputtering system equipped with a stoichiometric GST alloy target. The GST was then patterned using a lift-off process in a warm acetone bath. After that, the input and output waveguide and the pixelated region were defined by a second EBL and ICP. Finally, a 2 µm thick SiO${}_{2}$ layer was deposited on the whole of the device as an upper cladding layer by plasma-enhanced chemical vapor deposition (PECVD).

## 4. Conclusions

In conclusion, we have proposed an ultra-compact integrated optical switch based on PCM as a solution to meet the demands of future ultra-high density, reconfigurable, and scalable PICs. The device comprises GST nano-disk and a inverse-designed pixelated sub-wavelength structure. The device occupies a compact footprint of 0.9 µm × 1.5 µm. The phase of the PCM (a-GST and c-GST) controls the ON and OFF state of the optical switch. Our simulation results exhibit exhibit low IL of 0.45 dB, and high ER of 18.0 dB at 1550 nm. Additionally, we evaluated the effect of different process errors on device performance and proved that the design has a high degree of manufacturing tolerance. In general, these findings may provide a new approach to the miniaturization of optical switches and contribute to the development of datacenter networks and optical deep learning in the near future.

## Figures and Tables

**Table 1:** Bandwidth performance comparison between S1, S2 and S3.

	FOM	1550 nm		1535–1565 nm	
		IL (dB)	ER (dB)	IL (dB)	ER (dB)
**S1**	0.882	0.44	16.5	0.43∼0.48	16.5∼16.6
**S2**	0.887	0.45	18.0	0.44∼0.47	17.3∼18.6
**S3**	0.833	0.96	23.4	0.95∼1.00	23.1∼23.5

## Data Availability

Not applicable.
